# Supplementary material for: A computational modeling of pri-miRNA expression
Source: PLoS One. 2024 Jan 2;19(1):e0290768. doi: 10.1371/journal.pone.0290768 (PMC10760784; doi:10.1371/journal.pone.0290768)
Supplement: Attachment — Submitted filename: response_11182022.docx [file pone.0290768.s011.docx]

Dear Editor,

Thanks for the opportunity to submit this revision! We appreciate the reviewers for their valuable suggestions and comments. We have addressed all comments in the modified manuscript and provided additional materials.

Best Regards,

Xiaoman Li

Professor

Burnett School of Biomedical Science

University of Central Florida

The following is the point-by-point response to the reviewers' comments and concerns.

**Reviewer #1**:

**Comment 1**: What would be the biological significance of the model? Are the associated mRNA enriched in biological functions? Which ones? The text is missing a more detailed description of the results from the biological & functional perspective.

**Response:** Thanks for great questions and suggestions. We emphasized the significance in introduction and discussion. We provided the GO analysis results of miRNAs as an additional supplementary file S4. We also added some further descriptions of the biological significance of the model based on the GO enrichment analysis.

**Comment 2**: Another explanation why the results are significantly worse in the Cross Validation (CV) WRT the large model is lack of generalization. This should be at least discussed in the text. The lack of training and test should be addressed. Additionally even in CV it is highly recommended to exclude from the training, the samples in which the test predictions are made or very similar samples to the test set that are present in the training.

**Response**: Thanks for great comments! We agree that the correlation in the CV is not as high as that in the large model, which implies that the miRNA expression is condition-specific and the model trained from a subset of samples cannot fully explain the expression variation in new samples. We emphasized this condition-specific nature in the modified manuscript. Note that in both CV and the large model, the correlation is statistically significant (p-value 0). We also followed your suggestion to remove similar samples in testing that are used in training. We found that the trained model still can explain the pri-miRNA expression well, although not as well as the full model.

**Comment 3**: How is the performance of the model affected by the choice of the TSS neighborhood? Can the authors give a quantitative estimate of the variation in predicted and observed pri-miRNA correlation compared to the model described?

**Response**: Thanks for the comment! The performance of the model is not affected by the choice of the neighborhood sizes we used. We provided additional information in the first paragraph of page 15.

**Comment 4**: How many of the target mRNAs are present in the selected 2312 mRNAs? The authors conclude that the associated mRNAs are different from the target mRNAs, but they should report how many were excluded after their correlation filter and quantify the correlation of associated mRNAs and target mRNAs. Higher correlation of the associated mRNAs WRT target might be due to model overfitting. This should be discussed in the text.

**Response**: Thanks for the great comments! In the modified version, we reported the bynber if target mRNAs in the 2312 mRNAs in Supplementary Tables S5 and S8. We also calculated the hypergeometric testing p values and showed that the target genes are not enriched in the associated genes (Supplementary Table S8).

**Comment 5**: Related to the previous point, I would suggest an analysis of target genes enrichment across associated miRNAs of pri-miRNAs showing higher expression & larger variation WRT the rest of pri-miRNAs? Also, do these pri-miRNAs correlate better with their respective mature miRNAs?

**Response**: Thanks for the suggestion. We added the enrichment analysis as mentioned above. We found no significant difference between the two groups of pri-miRNAs based on enrichment analysis between target mRNAs and associated mRNAs. The pri-miRNAs showing higher expression & larger variation do not correlate better with their mature miRNAs (Table S10).

**Comment 6**: Does including genes that show less correlation than the threshold used (r=0.75) between CAGE & RNAseq increase noise in the model? Can the performance change be measured and discussed in the text?

**Response**: Thanks for the great comments! We chose this threshold (r=0.75) based on the false discovery cutoff 1. Because we want the trained model is applicable to both RNA-seq and CAGE data, it is necessary to use only those mRNAs that show consistent expression between the two platforms. With this said, including mRNAs with smaller correlation may introduce uncertainty to the model and prevent its application to the RNA-seq data or CAGE data.

**Comment 7**: finally I would encourage the authors to publish their model in a publicly accessible repository.

**Response**: Thanks for the suggestion. We provided the full model at <https://doi.org/10.6084/m9.figshare.21578847>.

**Reviewer #2**:

**Comment 1**: In Table S4, the miRNA nomenclature is non standard. the "star" (*) notation to denote passenger strands has been abandoned a long time ago and mature miRNAs are named after the portion (5p, 3p) of the corresponding hairpin they arise from. Furthermore, the targetScan_targets field is "N/A" for ~235 out of 278 miRNAs. However, most of these miRNAs "without targetScan tqargets" have a long list of targets predicted by targetScan. I suspect that automatic retrieval of targetScan targets failed (perhaps due to inappropriate nomenclature of mature miRNAs in the dataset by the authors). This casts a serious shadow on the claim that "These associated mRNAs differ from their corresponding target mRNAs" as in 85% of the cases the miRNAs targets are (mistakingly) missing, hence they of course do not overlap "associated genes". This part of the analysis should be repeated after fixing the issue with the missing targetScan targets.

**Response**: Thanks for the great comments! Sorry for the confusion about the miRNA star here. We would like to point out that the "star" (*) notation in our table is not used to denote the passenger strands, which should be following the miRNA names. Instead, we added the "star" (*) in front of the miRNA names to indicate the name of miRNAs in mirGeneDB previously and now removed them. Thanks for pointing out the issue with the TargetScan targets. We previously used the targets with the conserved sites and now we used both conserved and non-conserved sites by TargetScan to define the targets. Our conclusions still hold.

**Comment 2**: Figure quality can be dramatically improved in terms of resolution, labeling, clarity of legends, and, most importantly, the kind of data reported.

In Figure 1 in its current form the only take home message is that correlations assessed by Pearson or Spearman algorithms yield comparable values. However, the point here is how good are the observed correlations (compared to a putative null hypothesis). Therefore, comparing the distribution of correlation values obtained using LASSO modeling with mock data using a boxplot or violinplot would be more appropriate.

It is not entirely clear to me what is depicted in Fig 1A, did the authors compare the "real" values and "computed" values on the very same identical data (i.e. traning data == test data)? If this is the case, such validation is tautological and should be removed. Furthermore, what is the rationale for choosing 10-fold cross validation (over 5-fold cross validation or any other n-fold?). The number of samples (> 1800) is very large, a robust trend should be validated by simply splitting the dataset in two halves. n-fold cross validation is usually more appropriate when dataset size is limited.

In figure 2 only 2 example miRNAs are reported. A cumulative picture reporting the general trend for all miRNAs should be provided instead. Furthermore, mirTarBase and targetscan targets should be analysed separately. Alternatively a boxplot displaying side to side (1) the distribution of the median correlation with "associate genes" for each miRNA (2) the distribution of the median correlation with targetScan target genes for each miRNA (3) the distribution of the median correlation with mirTarBase target genes for each miRNA.

Figure 3 Rather than reporting the observed outcome for each of the 10-fold cross validations, an average (or median) +/- SD should be reported. Howevver, check my comment above about the choice of 10-fold cross validation over other validation strategies available.

**Response**: Thanks for the constructive comments! We updated the figures. For figure 1, we replaced it with box plots of correlations from ten two-fold validation experiments as you suggested. We revised the validation method. The new validation method is ten experiments of two-fold validation. We randomly choose half of the samples in each experiment as training and the other half as testing. In addition, we removed the same primary cell types, cell lines, or tissues used in training from the testing samples in each experiment. What we compared is the predicted expression in the testing data with the known expression in the testing data. We assessed the significance based on the asymptotic distribution of the correlation (t-distribution) as we have enough testing samples here. For figure 2, we used a violin plot to display three distributions side by as you suggested. We revised figure 3 due to the usage of the two-fold cross validation here. We also added additional description related to figure 3 in the manuscript.

**Comment 3**: The cutoff explored by the authors (a value > 0 in at least 20% of samples) for miRNA-TSS CAGE data will yield a zero inflated dataset. This is a relevant limitation of the study. Having a 0 in 80% of cases implies that any algorithm predicting always "0" will attain an 80% success. The authors should consider a more stringent cutoff to avoid such issues, restricting their analysis to the pri-miRNAs which exhibit a value > 0 in 80% of the cases. Otherwise, a strategy to cope with zero inflated data should be outlined and applied. Incidentally, 80% > 0 is the cutoff the authors apply to mature miRNA analysis. One might wonder why two such different cutoffs (20% > 0 for pri-miRNA and 80% > 0 for mature miRNAs have been applied in the same manuscript.

**Response**: Thanks for the great comments! We used the cutoff 80% in the entire manuscript now.

**Comment 4**: the claim that data reported in ref (31) are due to an error or misinterpretaion of the data by the authors i not supported by the data. While I totally agree with Li and colleagues as long as the point is that pri-miRNAs and the corresponding mature miRNAs display a rather poor correlation (and I believe this makes pri-miRNA quantification a very poor proxy for mature miRNA expression) in (31) Fig 3C de Rie et al have compared the distribution of correlations observed between CAGE-pri-miRNA data (median ~ 0.2 as assessed by the figure) with the ones observed after breaking ties in the data (randomly permuting CAGE-pri-miRNA data and corresponding mature miRNA data), an accepted method of mimicking the null hypothesis. de Rie et al make a formally correct statement that the amount of correlation they observe is significantly higher than the one expected by chance. Furthermore the numbers they observed match quite closely the ones reported by Li inhere (a 55 percentile of 0.201).

So de Rie et al are correctly reporting a statistically significant correlation between CAGE-pri-miRNA and mature miRNAs, and their data perfectly agree with the analysis reported inhere. If the authors feel that a median correlation of 0.2 (although statistically significant) may be of rather poor interest from a biological point of view, the discussio would be the right session of the manuscript to laborate on that, but a negative comment on de Rie et al in the results section (while reporting essentially the same numbers) to question their interptretation of the data is inappropriate.

I would also state here that I am not in any way associated with any of the authors of (31).

Response:

**Response**: Thanks for the great comments and unbiased assessment! We removed the negative comment on de Rie et al. We agree with your comments on the significance of the median correlation by de Rie et al. and the fact that there is no contradiction with our analysis. What we focused here is the individual miRNAs instead of the median correlation of all miRNAs. We measured the significance of the expression correlation of a pri-miRNA and its mature miRNA based on the asymptotic distribution and found that the majority of pri-miRNAs do not have a correlated expression with their mature miRNAs in the modified version. But we did remove the negative comments on de Rie et al.’s work.

**Comment 5**: The GO terms analysis is not reported, not even in the supplementary data. It would be interesting to see which are the GO terms associated with "associated genes" of each miRNA, whether they match the GO terms associated with their target (e.g. it would be conceivable that a set of regulators or transcription factors involved in apoptosis affect (and are hence associated with) the transcription of a mature miRNA targeting apoptosis related genes). Th authors should provide the full output of their GO terms analysis and elaborate on that.

**Response**: Thank you for pointing this out. We include Table S4 for GO terms associated with associated genes. We also have further discussions about the GO term results.

**Comment 6**: page 14 "or the three neighborhood sizes, the median number of associated mRNAs identified was 164, 163, and 164, respectively, indicating the robustness of the inferred associated mRNAs" This is not correct. The fact that using different neighborhood sizes does not affect the associated mRNAs is rather telling us that the neighborhood window has a poor effect on the assessment of pri-miRNA-CAGE, thus yielding consistent input data (regardless of the neighborhood size) to the same LASSO algorithm, which obviously results into very similar output. However, any conclusion on the robustness of the algorithm itself would be an over-interpretaion of this observation. The robustness of the approach could be validated instead by testing it independently on different datasets (or different slices of the same dataset).

**Response**: Thanks for the comment! We agree that what you point out could be an alternative explanation of the similar median number of associated mRNAs. As there is no much additional independent samples for testing, we removed the robustness statement.

**Comment 7**: In table S4 some miRNAs are repeated twice (e.g miR-101-1, miR-29b-2) and in some cases (miR-101-1) the TSS taken into account are almost completely overlapping.

**Response**: Thanks for pointing it out. Some miRNAs TSS are reported from different studies, and some of them are very close to each other. We would like to show if there are any differences in associate genes as the result of these TSS differences. After we focus our study with miRNAs expressed in 80% of the samples, only hsa-mir-374a, hsa-mir-1303, hsa-mir-545 have multiple occurrence. We found that different TSSs of these miRNAs do have different associated mRNAs. The table is now renamed as Table S5.

**Comment 8**: the datasets, miRNAs and samples analysed, although described in the text, should be described in tables depicting the number of samples, miRNAs and pri-miRNA CAGE fed into each analysis. GO terms output should also be provided in tables (and possibly representative examples reported as figures). The validation data about the LASSO approach should also be reported as supplementary tables (i.e. values underlying Fig 1).

**Response**: Thanks for the suggestions. We updated the supplementary data. The number of samples is indicated in supplementary file names. The GO terms output is now Table S4 and adding Figure 4 as representative examples. We attached the original data used for the previous figure 1 in Tables S6 and S7.

**Comment 9**: the authors should elaborate on the biological significance of inferring pri-miRNA expression based on a set of mRNAs. Why this finding is potentially interesting from a biological point of view? do they expect that "associate mRNAs" may encode for transcription factors, RNA binding enzymes or other proteins directly involved in promoting/repressing transcription of pri-miRNAs?

**Response**: Thanks for the suggestion. The significance to infer the pri-miRNA expression is that we cannot measure the pri-miRNA expression easily. But we can measure the expression of mRNAs. If we can model it through the expression of mRNA expression, it may provide a way to study pri-miRNA expression directly, especially in the RNA-seq experiments. We emphasized this point in the introduction and discussion. About the function of the associated mRNAs, we analyzed their function in Table S4.
