# Supplementary material for: A computational modeling of pri-miRNA expression
Source: PLoS One. 2024 Jan 2;19(1):e0290768. doi: 10.1371/journal.pone.0290768 (PMC10760784; doi:10.1371/journal.pone.0290768)
Supplement: Attachment — Submitted filename: response.docx [file pone.0290768.s012.docx]

**Reviewer #1**

**Comment 1**: Thanks to the authors for their replies & modifications to the text. Below my comments:

- Figures resolution should be highly improved.

**Response**: Thanks for the comments! We updated the figures to increase the font sizes and resolution. The figures especially fig, 4 may still look vague in the pdf because of the plos one online submission conversion. But if you click the blue links on the top of the pdf pages that contain the figures, you can see the high resolution figures.

**Comment 2**: In Figure 2, I noticed that some of the associated mRNAs show correlation of 1 with the pri-miRNA. Can the authors resolve the potential concern that these mRNAs are identical to the modeled pri-miRNAs? If removed, how would this affect the performance of the model? This raises potential concerns of contamination between input and predicted variables, that if confirmed, the authors should address by comparing the distribution of performance across pri-miRNA with the performance after removing those putatively identical data points to the predicted values.

**Response**: Thanks for the great comments! Among the 195 pri-miRNAs we considered in this study, there are 3 pri-miRNAs with their Pearson’s correlation coefficient equal to 1 with one and only one of their associated mRNAs. In total, there are 14 pri-miRNAs with one and only one associated mRNA having their TSSs within +/-100 base pairs of the corresponding pri-miRNA TSS. We removed these associated mRNAs and retrained the model when we considered the expression of these pri-miRNAs. We found that the minimum, mean, and median Pearson’s correlation of the predicted and true expression per miRNA was 0.78, 0.91 and 0.92, respectively. The minimum, mean and median Spearman’s correlation per miRNA was 0.27, 0.82 and 0.83, respectively. When measured the similarity of the predicted expression in every sample, the minimum, mean, and median Pearson’s correlation per sample was 0.45, 0.87 and 0.90, respectively. The minimum, mean and median Spearman’s correlation per sample was 0.39, 0.83 and 0.85, respectively. Based on the almost identical correlation to the correlation with the original model we presented, it is safe to say the mRNAs with similar TSS with pri-miRNA should not be a concern in this study. We modified the paper correspondingly.

**Reviewer #2**

**Comment 1**: The authors have only partially addressed my comments. Overall, conclusions are not supported by the data as key controls are still lacking.

- In particular, Fig 1, which underlies tha main finding of the paper is still lacking a "negative control" (I had suggested comparing correlations observed using the LASSO model to some kind of mock data, Comment #2 of my review).

**Response**: Thanks for the feedback. Sorry that we thought we addressed this issue with the asymptotic distribution of the correlation and did not use the negative controls previously. We now added two different negative controls. For the negative control #1, we randomly permute each mRNA expression across the 1829 samples while keep the same pri-miRNA expression to see how well the pri-miRNA expression can be modeled by these randomized mRNA expression data. For the negative control #2, we randomly assign a mRNA expression by permuting the expression of all mRNAs in one sample so that overall expression of a mRNA across the 1829 samples becomes random. We then ran the same analysis to model the pri-miRNA expression. We compared the correlation of the predicted pri-miRNA expression and the true pri-miRNA expression from the two negative control datasets with the correlation obtained from the original data in the manuscript. See the comparison in the following figure (with a model based on all 1829 samples) and figure 1 (with a model based on the two-fold cross-validation) in the paper. The correlation from the original data is much larger than that from the negative control data.

**Comment 2:** Furthermore, Fig 2 is now reporting essentially identical plots for "associate" vs "target" mRNAs, with the notable exception of a peak centered on Correlation =1. However the statistical (and biological) significance of Pearson (or Spearman) values > 1 (clearly depicted in Fig2) is questionable... how could the authors possibly obtain values > 1? and if they did not, which kind of "smoothing" algorithm is resulting in such an artifact?

**Response:** Thanks for pointing out this mistake. The correlation value was not larger than 1. We used the Python Seaborn package to plot the violin plot, which features a kernel density estimation of the underlying distribution. This is the reason why the distribution seems >1 but there’s actually no correlation >1. We have redrawn the figure 2 in the current version. We also removed the mRNAs with their TSSs within +/-100 base pairs of the pri-miRNA TSS when modeling the expression of this pri-miRNA. Our conclusions still hold.

**Comment 3**: The data reported in Table S7 (which does not match the description reported in the manuscript) report identical data regardless of the use of De Rie et al data or the author's data. Again, this further reinforces the opinion by this reviewer that any divergence between De Rie et al and the authors of this manuscript pertains the interpretation of the data, not the results. Therefore the Results paragraph dedicated to this issue is pointless.

**Response:**  Thanks for the comments! We are not sure we precisely understand your points here. Table S7 is about how well the predicted pri-miRNA expression correlates with the actual pri-miRNA expression per sample, which has no relation with De Rie et al.’s point that the expression of pri-miRNAs and mature miRNAs correlates well. In other words, Table S7 is about the modelling of pri-miRNA expression with mRNAs, which does not deal with mature miRNA expression. De Rie et al. did not model pri-miRNA expression in their study. In terms of the results, the modeling of pri-miRNA expression by mRNA expression is not in De Rie et al.’s study, which is the main purpose of our study. Moreover, about pri-miRNA and mature miRNA expression correlation, our results are different from De Rie et al.’s as well. We showed that the majority pri-miRNAs do not have correlated expression with their mature miRNAs for individual miRNAs (FDR<0.01), which is supported by our data and De. Rie et al.’s data. De Rie et al. showed the AVERAGE expression correlation between pri-miRNAs and their mature miRNAs is significant and concluded that the pri-miRNAs have a correlated expression with their mature miRNAs. In fact, more than half of the pri-miRNAs do not correlate well with their mature miRNAs (Supplementary Table S9).

**Comment 4**: The GO term analysis relies on FDR <1. This cutoff is meaningless, as only excludes tests yielding a FDR=1 while retaining anything with an FDR = 0.9 or 0.8 which are obviously unreliable findings. Furthermore the FDR for each test is not reported (only P-values are available). Therefore, no conclusion can be drawn on this analysis.

**Response:** Thank you for pointing this out! We agree that the FDR cutoff should be smaller. We have changed the FDR cutoff to 0.1. We also report the FDR together with p-values.
