# Supplementary material for: A computational modeling of pri-miRNA expression
Source: PLoS One. 2024 Jan 2;19(1):e0290768. doi: 10.1371/journal.pone.0290768 (PMC10760784; doi:10.1371/journal.pone.0290768)
Supplement: Attachment — Submitted filename: Response_05212023.docx [file pone.0290768.s013.docx]

Dear Dr. Manuela Helmer-Citterich,

We appreciate the comments from the reviewer and sincerely thank the reviewer for the time and the comments. We have edited our manuscript to clarify some points which were not described clearly or properly previously, and also added some new materials. We hope the modified manuscript can be accepted.

Best Regards,

Xiaoman Li

Professor

University of Central Florida

We now address the points raised in a point-by-point fashion.

**Reviewer #2**:

**Comment 1:** The authors have addressed comment 1, but several inconsistencies between data in supplementary Tables and claims in the text and figures remain.

The authors have fully addressed my comment.

**Response:** We appreciate your feedback and are glad we could address your concern related to comment 1. We will respond to the inconsistencies you mentioned below in the following sections.

**Comment 2:** The authors have simply cut the y axis at y=1. Obviously, values aove 1 disappeared. This is not acceptable. Furthermore, the copy of Fig 2 reported in the author's response is different from the one reported in the text, and both differ from the one which was in the previous version.

If values > 1 are not in the original data but are the result of a smoothing function (kernel density) this should be documented. Nevertheless, my original question still holds: what is the meaning od Pearson correlation =1 in this plot? How do the authors explain this extreme value? which pairs do yield such astonishingly high correlations? Does this extreme result stem from pri-miRNA:mRNA pairs which, perhaps due to duplicated annotations, actually represent the same RNA molecule (or two largely overlapping RNA moleculesl? This must be investigated picking those pairs and checking annotation, the simple claim that RNAs with TSS within 100 nt of the pri-miRNA TSS have been removed is not sufficient.

**Response:** Thank you for the feedback!

Here is the reason that we cut the y-axis at y=1 in the plot. As we mentioned before, the kernel density estimation caused the initial figure in the first submitted version to seem to have values >1, although there were no value >1. To avoid this confusion, we adjusted the plot accordingly in the last revision.

The correlation = 1 means that there is indeed an overlap of The TSS regions of primary miRNAs and their associated genes. As we mentioned in the previous response, among the seventeen miRNA-mRNA pairs with their TSSs close to 100 base pairs, three pairs have a correlation value 1 and five pairs have a correlation value close to 1. We removed these primary miRNA and mRNA pairs when we predicted the primary miRNA expression in the last version. However, we did include these pairs in other analyses in the last version, which resulted in certain pri-miRNA-mRNA pairs with correlation 1 in Fig. 2. To effectively address the issue caused by miRNA-mRNA pairs with close TSS regions, we removed all these pri-miRNA-mRNA pairs to perform the lasso analysis and updated the lasso model and the entire manuscript this time.

Regarding the figures, we want to emphasize that there is no inconsistency between the figures provided in the manuscript and our response. The figure we included in our previous response was based on two different negative control datasets you suggested, which we pointed out in the previous response letter. The Fig. 2 in the previous manuscript compares the expression correlation of the primary miRNAs and their associated genes with the expression correlation of the pri-miRNAs and their miTarBase and TargetScan target genes. The two figures are thus supposed to be different. We did not include the figure in the response in the manuscript because the Fig. 1 in the previous manuscript already described the same analysis with two negative control datasets in a different format. We apologize for any confusion that may have arisen and hope that this clarifies the matter.

**Comment 3:** Table S7 is enitled "Table_S7_The_Pearsons_and_spearmans_correlation_coefficient_between_pri-iRNAs_and_mature_miRNAs". Furthermore, the reported values (column 1) have an average value of 0.17, which is at the odds with the correlation values reported in Fig 1. If Table S7 reports (as the authors wrote in their reply) how well the predicted pri-miRNA expression correlates with the actual pri-miRNA expression per sample (which are supposed to be the correlation values undelying Fig 1, blue boxplot, average~0.6), there is a clear inconsistency in the data, as the average value in Table S7 is 0.17. I therefore suspect that, in line with the actual title of the Table S7 and the column names (pearson_corr_based_on_our spearman_corr_based_on_our pearson_corr_based_on_derie spearman_corr_based_on_derie) this Table reports on the miRNA:pri-miRNA correlation using two different dataset (stemming actually from the same experiment) and again these data further confirm that miRNAs display a minor yet significant correlation with pri-miRNAs in both datasets.

**Response:** Thank you for your comment. Regarding your concern about the relationship between Figure 1 and Table S9 (previously S7), we would like to clarify that they represent different analyses and are not directly comparable. Figure 1 shows the results of ten two-fold cross-validation experiments for predicting pri-miRNA expression, which calculated the correlation between the true expression and the predicted expression of pri-miRNAs, with comparison to two different negative control datasets. On the other hand, Table S9 (previously S7) shows the correlations between pri-miRNA expression and mature miRNA expression. Pri-miRNA expression is different from the mature miRNA expression. The two results are thus not directly connected and should not be compared against each other. Furthermore, we want to emphasize that our analysis in Table S9 shows that about half of the pri-miRNAs do not correlate well with their mature miRNAs. This highlights the importance of analyzing each individual pri-miRNA, rather than looking at the overall average.

**Comment 4:** In Table S4 FDR is not reported, only P-values are reported. Furthermore, while data in Table S4 have been computed for each single miRNA, Fig 4 does not report a specific miRNA, so it is unclear how those GO terms were obtained.

**Response:** Thank you for taking the time to review our work and provide feedback. We would like to respectfully clarify that the FDR q-value for each GO term was reported in Table S4. Moreover, we would like to emphasize that Fig 4 does indeed report the GO results based on the function of the associated genes of a specific miRNA, hsa-miR-92a, as stated in the figure legend.
