# Supplementary material for: A computational modeling of pri-miRNA expression
Source: PLoS One. 2024 Jan 2;19(1):e0290768. doi: 10.1371/journal.pone.0290768 (PMC10760784; doi:10.1371/journal.pone.0290768)
Supplement: Attachment — Submitted filename: response_07212023.docx [file pone.0290768.s014.docx]

Dear Dr. Manuela Helmer-Citterich,

We appreciate your help to identify the wrong links. We also thank the reviewer for the careful review and helpful suggestions. We have edited our manuscript to correct these links and added more information. We hope the modified manuscript can be accepted.

Best Regards,

Xiaoman Li

Professor

University of Central Florida

We now address the points raised in a point-by-point fashion.

**Reviewer #2**:

**Comment 1:** In these third revision as well as in the second revision, although the text reported for the figshare link for Supplementary tables has been updated ("21578847.v5"), the underlying hypertext link is still pointing to v1 (https://figshare.com/articles/online_resource/A_computational_modeling_of_pri-miRNA_expression/21578847/1), for this reason I could not access the appropriate version of the Supplementary Tables during second round of revision, thus explaining why I saw inconsistencies between the Tables published on figshare and the text. The link should be updated.

**Response:** Thank you so much for helping us to correct the links. Now they are updated.

**Comment 2:** I still have concerns regarding some of the statements in section "The expression of the majority of mature miRNAs do not correlate

well with the expression of pri-miRNAs", for example:

"with a 57.71 percentile of 0.1592" is a really odd statement, why should one actually choose the "57.71th percentile" of a distribution? Does this value perform any better than the median (50 percentile) which is commonly used instead?

**Response:** Thank you for the feedback! As we stated in the paper, it is the cutoff with the FDR of 0.01. We also added this information at another place in the text in the modified version.

**Comment 3:** Finally, the authors should acknowledge somewhere that the values reported in Table S9 show that correlation coefficients computed here and in de rie are in fact identical in 154/176 miRNAs, and when they differ, in most cases the difference is negligible (e.g. miR-23a: 0.609994705145189 vs 0.609835708540695) so the point here is entirely on data interpretation, not on the data.

**Response:** Thank you for your comment. We added two sentences in the text to acknowledge that these correlation coefficients are quite similar to those in the previous studies, and the difference of our conclusion is based on different aspects of consideration of individual miRNAs, not all miRNAs together.
