# Supplementary material for: A computational modeling of pri-miRNA expression
Source: PLoS One. 2024 Jan 2;19(1):e0290768. doi: 10.1371/journal.pone.0290768 (PMC10760784; doi:10.1371/journal.pone.0290768)
Supplement: Attachment — Submitted filename: response_08132023.docx [file pone.0290768.s015.docx]

Dear Dr. Manuela Helmer-Citterich,

Thanks for the opportunity to make this right. We have edited our manuscript to clarify the procedure to control the false discovery rate. We hope the modified manuscript can be accepted.

Best Regards,

Xiaoman Li

Professor

University of Central Florida

We now address the points raised in a point-by-point fashion.

**Editor Comments**:

**Comment 1:** After so many revisions, I ask the Authors to consider the comments of the Reviewer to their last revision and make the requested amendments.

**Response:** Thanks for the suggestion. Yes, we revisited the reviewer comments from the last revision and provided the requested details in the modified version.

**Reviewer #2**:

**Comment 1:** The methods section does not provide details on how the "FDR of 0.01" was calculated. This should be explained clearly. It is also unclear the statement "with a 57.71 percentile of 0.1592 (FDR<0.01)", the FDR reported is linked to a test about the likelyhood that the 57.71th percentile is 0.1592 or is simply the FDR associated with the corrlation observed for the pri-miRNA ranking at the 57.71th percentile.

**Response:** Thanks for the feedback! The FDR was calculated with the well-known Benjamini Hochberg algorithm. In brief, we calculated the p-value for the correlation of the pri-miRNA expression and its mature miRNA expression based on the asymptotic distribution of the correlation for each pri-miRNA. We then ranked the pri-miRNAs based on their p-values from the smallest p-value to the largest p-value. Finally, we checked whether the sum of the p-values from the first pri-miRNA to the current i-th one was larger than 0.01 for i starting from 1 and stopped when the first sum was larger than 0.01. When we stopped at this specific i-th pri-miRNA, we reported the top (i-1) miRNAs as significantly correlated pri-miRNAs with FDR smaller than 0.01. In our case, this i-th miRNA had a correlation of 0.1592 (the larger the correlation, the smaller the p-value), which corresponding to the 57.71-th percentile of the correlations. We modified the paper to include a brief description of this standard procedure.
